# A Lead-μ_2_-Tetrylide Complex with Osmium(IV) Terminal Components

**DOI:** 10.1021/acs.inorgchem.4c02520

**Published:** 2024-08-05

**Authors:** Javier A. Cabeza, Miguel A. Esteruelas, Israel Fernández, Susana Izquierdo, Enrique Oñate

**Affiliations:** † Departamento de Química Orgánica e Inorgánica, Centro de Innovación en Química Avanzada (ORFEO-CINQA), Universidad de Oviedo, 33071 Oviedo, Spain; ‡ Departamento de Química Inorgánica, Instituto de Síntesis Química y Catálisis Homogénea (ISQCH), Centro de Innovación en Química Avanzada (ORFEO-CINQA), Universidad de Zaragoza, CSIC, 50009 Zaragoza, Spain; § Departamento de Química Orgánica I, Centro de Innovación en Química Avanzada (ORFEO-CINQA), Facultad de Ciencias Químicas, Universidad Complutense de Madrid, 28040 Madrid, Spain

## Abstract

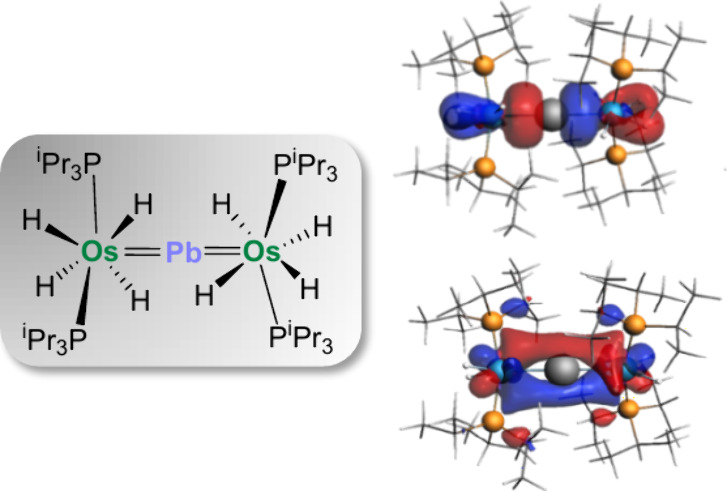

A bare lead atom is a σ-donor ligand capable of linearly bonding and stabilizing two units of a classical polyhydride complex, with a high-valent metal center. As a proof of concept, we have prepared and characterized the μ_2_-tetrylide complex (P^i^Pr_3_)_2_H_4_Os=Pb=OsH_4_(P^i^Pr_3_)_2_ in the reaction of OsH_6_(P^i^Pr_3_)_2_ with Pb{N(SiMe_3_)_2_}_2_. Although the Pb–Os bonds exhibit electrostatic interaction, the main orbital interactions result from two dative σ bonds from the lead atom to the osmium centers. The latter also provide much weaker π-backdonations.

## Abstract 2

A bare lead atom is a σ-donor ligand capable of linearly bonding and stabilizing two units of a classical polyhydride complex, with a high-valent metal center. Accordingly, this Communication shows the preparation of the μ^2^-tetrylide (P^i^Pr_3_)_2_H_4_Os=Pb=OsH_4_(P^i^Pr_3_)_2_ complex, through the reaction of OsH_6_(P^i^Pr_3_)_2_ with Pb{N(SiMe_3_)_2_}_2_, and analyzes its Os−Pb bonds.

μ_2_-Tetrylide complexes challenge the current limits of molecular coordination chemistry. They are formed by a bare atom of a Group 14 element, which is located in the center between the low-valent metal ions of two identical unsaturated transition-metal compounds; L_*n*_M=E=ML_*n*_ (E = C, Si, Ge, Sn, Pb). Only a small number of compounds have been characterized.

Carbide derivatives are the only ones that have a moderately significant representation,^1^ which includes complexes of rhenium,^2^ iron,^3^ ruthenium,^4^ and rhodium.^5^

The reported complexes of silicon are rare, and their tetrylide character is sometimes controversial. In 2018, Filippou’s group reported that a two-electron reduction of molybdenum compound Tp′(CO)_2_Mo≡Si–Mo(CO)_2_(PMe_3_)Tp′ [Tp′ = κ^3^-*N*,*N*′,*N*″-hydridotris(3,5-dimethylpyrazolyl)borate] with potassium graphite produces the dianion [Tp′(CO)_2_Mo=Si=Mo(CO)_2_(PMe_3_)Tp′]^2–^.^6^ More recently, Tilley and co-workers have observed that cobalt-promoted activation of SiH_4_ bonds generates tetrahydride–dicobalt–silicide species,^7^ although an AIM analysis locates two bond paths connecting silicon and two hydrides.^7b^

The first germanide species were discovered by the groups of Weiss and Hermann, between 1981 and 1985. Starting from Mn(η^5^-C_5_R_5_)(CO)_3_, they isolated (η^5^-C_5_R_5_)(CO)_2_Mn=Ge=Mn(CO)_2_(η^5^-C_5_R_5_) (C_5_R_5_ = C_5_H_4_Me, C_5_H_5_, C_5_Me_5_), after an acetic acid-promoted GeH_3_ dehydrogenation.^8^ The family of manganese compounds has recently been augmented by the complex (dmpe)_2_HMn=Ge=MnH(dmpe)_2_, reported by the Emslie group.^9^ In 2021, Arnold and co-workers crystallographically characterized the dirhenium germanide (η^5^-C_5_H_5_)(BDI)Re=Ge=Re(BDI)(η^5^-C_5_H_5_), which was prepared by reacting GeCl_2_·dioxane with Na[Re(η^5^-C_5_H_5_)(BDI)] [BDI = κ^2^-*N*,*N*′-bis(2,6-diisopropylphenyl)-3,5-dimethyl-β-diketiminate], at −78 °C.^10^

The situation with tin and lead is similar. Between 1985 and 1989, Herrmann and co-workers observed that the reactions of Mn(η^5^-C_5_Me_5_)(CO)_3_ with SnH_4_ and Mn(η^5^-C_5_H_5_)(CO)_3_ with PbCl_2_ led to (η^5^- C_5_Me_5_)(CO)_2_Mn=Sn=Mn(CO)_2_(η^5^-C_5_Me_5_) and (η^5^-C_5_H_5_)(CO)_2_Mn=Pb=Mn(CO)_2_(η^5^-C_5_H_5_), respectively, although only the latter was characterized by X-ray diffraction analysis.^11^ In 1992 and 2015, compounds [Bu_4_N]_2_[Pb{Pt(C_6_F_5_)_4_}_2_]^12^ and Pb{Pt(C_6_F_5_)_2_(bzq)}_2_ (bzq = 7,8-benzoquinolyl)^13^ were reported. Although they show a linear Pt–Pb–Pt arrangement, both X-ray diffraction analysis and NMR spectroscopy reveal a high coordination number for the lead atom, which is provided by the *o*-fluorine substituents of C_6_F_5_.

A recent theoretical study on the bonding situation in complexes (η^5^-C_5_H_5_)(CO)_2_Mn=E=Mn(CO)_2_(η^5^-C_5_H_5_) (E = C, Si, Ge, Sn, Pb) identifies mutually orthogonal π-delocalized systems along the linear backbone. The strength of the Mn–E bond decreases down the group and is accompanied by a lower contribution of the E atom ns valence orbital to the bond. This atom acts as σ donor and π acceptor, according to the Dewar–Chatt–Duncanson bonding model.^14^ On the basis of this feature of the heavier atoms of Group 14, we reasoned that the terminal L_*n*_M components of μ_2_-tetrylide complexes linked by such atoms should not necessarily carry a low-valent metal center; on the contrary, they would exhibit greater stability with terminal components of high-valence metallic centers. These metal centers have weak π-donor capacity, while they need σ-donor ligands to maintain their high valence.

Transition-metal polyhydride complexes typically involve metals in high oxidation states. Therefore, unsaturated members of this family should be excellent candidates to act as terminal L_*n*_M components, with the heaviest Group 14 elements. Furthermore, they are mild reducing agents,^15^ with reduction of the tetrylide source being the usual procedure for the preparation of the scarce μ_2_-tetrylide compounds characterized to date. Complex OsH_6_(P^i^Pr_3_)_2_ (**1**) is a prototypical polyhydride of the platinum group metals^16^ and a cornerstone in the development of osmium organometallics due to its rich stoichiometric^17^ and catalytic^18^ reactivity and its involvement in materials science as a precursor to osmium phosphorescent emitters.^19^ At temperatures above 50 °C, it loses H_2_ to give the unsaturated tetrahydride OsH_4_(P^i^Pr_3_)_2_, which has been trapped with 2e^–^ Lewis bases.^20^ These precedents led us to use it to test our hypothesis and, concurrently, prepare a completely different μ_2_-tetrylide complex. Tetrylene Pb{N(SiMe_3_)_2_}_2_ was used as the lead source.^21^

Reaction of the polyhydride with the tetrylene, in toluene, at 80 °C, for 22 h produced the precipitation of metallic lead as a fine dark solid and the formation of the desired complex (P^i^Pr_3_)_2_H_4_Os=Pb=OsH_4_(P^i^Pr_3_)_2_ (**2**), according to Scheme 1. After filtration over Celite, evaporation of the resulting solution, and purification of the crude solid by crystallization from pentane, at −30 °C, complex **2** was isolated as brown crystals in ≈60% yield. The H_2_ released by **1** reduces tetrylene to Pb and amine. Half of the Pb generated traps the unsaturated osmium tetrahydride, resulting from the dissociation of H_2_, to form **2**. Consistent with this, we also observe that tetrylene decomposes to Pb and amine in an H_2_ atmosphere.

**Scheme 1:**
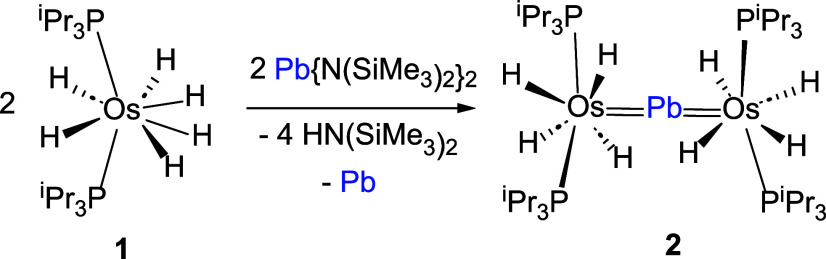
Formation of **2**

Complex **2** was characterized by X-ray diffraction analysis. The molecule (Figure 1) has the expected linear Os–Pb–Os backbone (exactly 180.0°) on a *C*_2_ axis. The coordination polyhedron around each osmium can be rationalized as a pentagonal bipyramid with the phosphines in apical positions (P–Os–P = 161.54(4)° and 161.32(5)°). Hydrides are found in the base along with the lead atom. The latter is very far from the hydrides (>2.56(4) or 2.64 Å in the density functional theory (DFT)-optimized structure).^22^ The absence of any Pb–H interaction was further confirmed by an AIM approach. Hydrides are of classical nature showing separations between them greater than 1.80 Å (DFT-optimized structure), in accordance with a strong σ-donor capacity of the lead atom.^23^ Thus, the general structure can be described as two pentagonal bipyramids, rotated relative each other 59.93(3)°, which have a common vertex where the lead atom is located. The Os–Pb distances are almost identical, 2.5842(3) and 2.5895(3) Å. Because the point group symmetry of the molecule is *D*_2_, the bipyramids are equivalent. Consequently, the ^31^P{^1^H} NMR spectrum shows a singlet at 55.7 ppm, corresponding to the phosphines, which splits into a quintuplet under off-resonance conditions due to the presence of four hydrides at each metal. Although the hydride ligands are inequivalent in the bipyramids, the room temperature ^1^H NMR spectrum contains only one triplet (^2^*J*_H–P_ = 14.3 Hz) at −8.07 ppm, consistent with the typical position exchange process exhibited by osmium polyhydrides in solution.^20^ The exchange occurs even at low temperature; only at temperatures below 165 K, two broad signals are observed. As expected for the classical nature of the polyhydride, a 400 MHz *T*_1_(min) value of 239 ms was obtained for this resonance, in methylcyclohexane-*d*_14_, at 253 K. Complex **2** is certainly stable. Its high stability even allows the obtainment of its high-resolution mass spectrum by electron electrospray ionization, without loss of any hydride ligands ([M]^+^; *m*/*z* 1240.5099), something highly unusual for polyhydride complexes.

**Figure 1:**
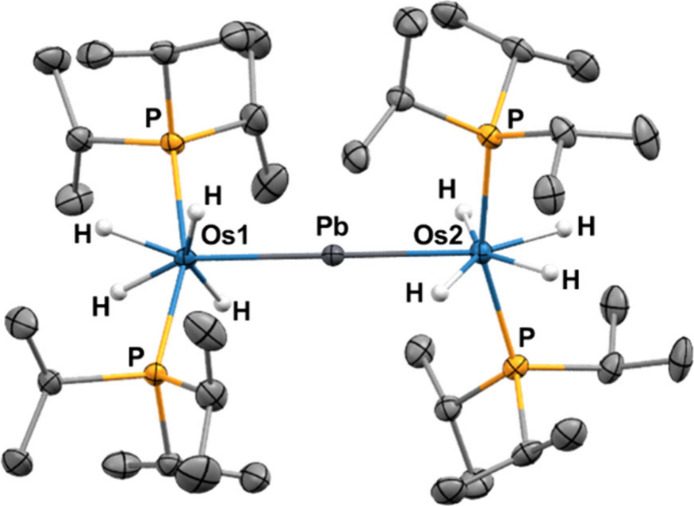
Molecular diagram of complex **2** (50% probability ellipsoids). Hydrogen atoms (except hydride ligands) are omitted for clarity.

The novelty of **2** and the need to understand its stability prompted us to analyze the Os–P bonds. To this end, DFT calculations at the relativistic dispersion-corrected ZORA-BP86-D3/TZ2P//RI-BP86-D3/def2-TZVP level were carried out. The optimized geometry matches the X-ray structure quite well, in particular the Pb–Os bond lengths (2.60 Å). Inspection of the occupied molecular orbitals involving the metal atoms (Figure 2) resembles that of the complex (η^5^-C_5_H_5_)(CO)_2_Mn=Pb=Mn(CO)_2_(η^5^-C_5_H_5_).^14^

**Figure 2:**
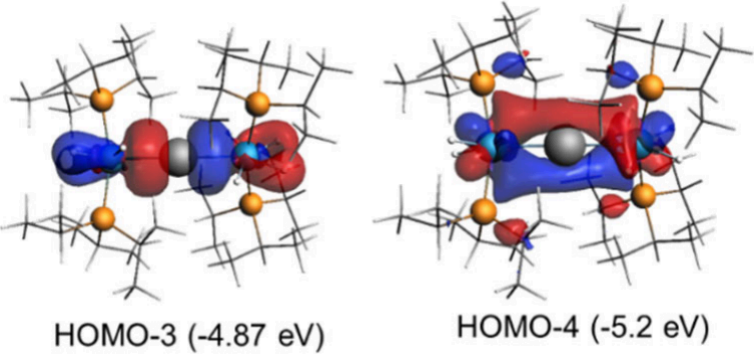
Selected computed molecular orbitals (isosurface value of 0.05 au) of complex **2**.

A more quantitative insight into the nature of the Pb–Os bonds was gained by applying the energy decomposition analysis (EDA) method. Explicitly, we investigated the interaction between (P^i^Pr_3_)_2_H_4_Os and PbOsH_4_(P^i^Pr_3_)_2_ fragments in the two possible bonding situations, namely: (i) a dative bond, thus involving singlet fragments, and (ii) an electron-sharing bond, therefore using fragments in their triplet state. As is well-known, the calculation that gives the smallest absolute value of the orbital term Δ*E*_orb_ corresponds to the most reasonable description of the bond, since then the formation of such a bond produces the smallest change in the electronic structure of the fragments.^24^ From the data in Table 1, it becomes evident that dative bonding represents the best description of the bonding in **2**.

**Table 1:** EDA (Energy Values in kcal/mol) of Complex *2*

	dative bond (singlet + singlet)	electron-sharing (triplet + triplet)
Δ*E*_int_	–82.1	–151.6
Δ*E*_Pauli_	185.8	268.8
Δ*E*_elstat_	–149.5	–200.2
Δ*E*_orb_	–87.9	–189.7
Δ*E*_orb_(ρ_1_)	–55.6	–54.0
Δ*E*_orb_(ρ_2_)	–11.7	–4.7
Δ*E*_disp_	–30.5	–30.5

The natural orbital for chemical valence (NOCV) extension of the EDA method was next applied to identify and quantify the main orbital interactions contributing to the total Δ*E*_orb_ term. From the data of Table 1, two main orbital interactions characterize the Pb–Os bond: a σ donation from a lone pair of the lead atom, mainly located at the p_*x*_ atomic orbital, to a vacant d atomic orbital of the osmium center [LP(Pb) → d_*x*^2^–*y*^2^_(Os)], denoted as ρ_1_, and a π-backdonation from a doubly occupied d atomic orbital of the osmium center to the vacant p_*z*_ atomic orbital of the lead atom [d_*xz*_(Os) → p_*z*_(Pb)], denoted as ρ_2_ (Figure 3). According to the computed stabilizing energies, Δ*E*(ρ), the σ-donation clearly dominates over the π-backdonation, which is comparatively much weaker. Therefore, from the point of view of orbital interaction, the bonding situation involving the Os–Pb–Os backbone of **2** can be summarized as follows: a bare lead atom establishes two strong dative σ bonds with two osmium centers, using its two available lone pairs. At the same time, the osmium centers provide weaker π-backdonations to the vacant p_*z*_ atomic orbital of the lead atom. This situation resembles that described for the complexes (η^5^-C_5_H_5_)(CO)_2_Mn=E=Mn(CO)_2_(η^5^-C_5_H_5_) (E = heavy element of Group 14) but sharply contrasts with that found in plumbylones, where the lead atom maintains its lone pairs to act as an electron acceptor.^25^ On the other hand, it should be noted that the orbital interaction is not the main contributor to the bonding. According to the data in Table 1, the Pb–Os bond exhibits a strong electrostatic interaction (≈56% of the total attractions), which is not surprising according to the significantly different computed NBO charges of the involved atoms: +1.19e for Pb and −1.16e for Os.

**Figure 3:**
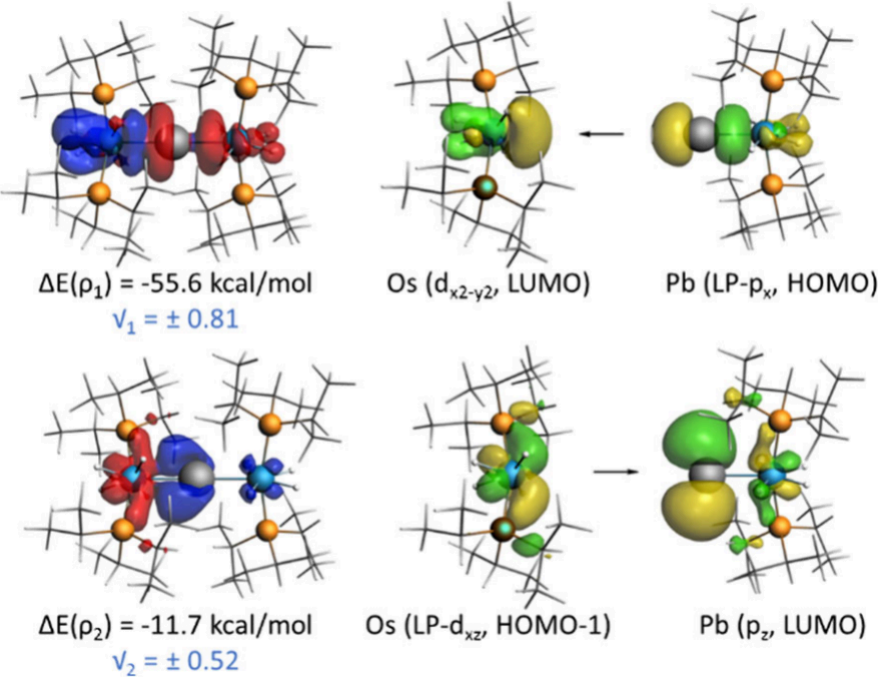
Contour plots of the main NOCV deformation densities ρ (isosurface value of 0.001 au), associated energies Δ*E*(ρ) and interacting orbitals compound **2**. The electronic charge flows from red to blue. The eigenvalues |ν| indicate the relative size of the charge flow.

The classical or nonclassical nature of the transition-metal polyhydride complex is determined by the basicity of the metal center, which is governed by the electron-donating capacity of its ligands. Electron-donating groups favor classical polyhydrides, while those with good acceptor properties benefit nonclassical interactions.^17,26^ The preparation of **2** containing a classical osmium(IV) tetrahydride as terminal components allows us to conclude that a bare lead atom is a strong σ-donor ligand in coordination chemistry, strong enough to be able to join and stabilize two units of a classical polyhydride complex in a linear manner. This finding also demonstrates that μ_2_-tetrylide complexes can contain two identical unsaturated terminal components of a high-valent metal center, in contrast to what has been observed so far.

## Supporting Information Available

General information, structural analysis of complex **2**, computational data, and NMR and IR spectra (PDF)
Cartesian coordinates of computed complex (XYZ)

## Supplementary Material

ic4c02520_si_001.pdf

ic4c02520_si_002.xyz
