# Supplementary material for: A Lead-μ2-Tetrylide Complex with Osmium(IV) Terminal Components
Source: Inorg Chem. 2024 Aug 5;63(34):15563–7. doi: 10.1021/acs.inorgchem.4c02520 (PMC11733942; doi:10.1021/acs.inorgchem.4c02520)
Supplement: Supplementary file 1 — ic4c02520_si_001.pdf [file ic4c02520_si_001.pdf]

SUPPORTING INFORMATION

A Lead-μ^2-Tetrylide Complex with Osmium(IV) Terminal Components

Javier A. Cabeza,[a] Miguel A. Esteruelas,[b,‡] Israel Fernández,[c] Susana Izquierdo,[b] Enrique Oñate[b]

[a] Departamento de Química Orgánica e Inorgánica, Centro de Innovación en Química Avanzada (ORFEO-CINQA), Universidad de Oviedo, 33071 Oviedo, Spain

[b] Departamento de Química Inorgánica, Instituto de Síntesis Química y Catálisis Homogénea (ISQCH), Centro de Innovación en Química Avanzada (ORFEO-CINQA), Universidad de Zaragoza-CSIC, 50009 Zaragoza, Spain

[c] Departamento de Química Orgánica, Centro de Innovación en Química Avanzada (ORFEO−CINQA), Facultad de Ciencias Químicas, Universidad Complutense de Madrid, 28040 Madrid, Spain

*Corresponding author's e-mail address: M.A.E.: maester@unizar.es.

Contents:

- Experimental Details	S2
- Structural Analysis of Complex **2**	S3
- NMR spectra	S4
- IR	S6
- Theoretical Calculations	S7
- References	S8

Experimental Details:

General Information

All reactions were carried out with exclusion of air using Schlenk-tube techniques or in a drybox. Pentane and toluene were obtained oxygen- and water-free from an MBraun solvent purification apparatus, and then were dried over activated 4Å sieves at less for two days. ^{1}H, $^{13}C\{^{1}H\}$ and $^{31}P\{^{1}H\}$ NMR spectra were recorded on Bruker Avance 300 MHz or Bruker Avance 400 MHz. Chemical shifts (expressed in ppm) are referenced to residual solvent peaks. Coupling constants J and N ($N = J_{P\text{-}H} + J_{P'\text{-}H}$) are given in hertz. C, H, and N analyses were carried out in a PerkinElmer 2400 CHNS/O analyzer. High-resolution electrospray mass spectra were acquired using a MicroTOF-Q hybrid quadrupole time-of-flight spectrometer (Bruker Daltonics, Bremen, Germany). $OsH_6(P^iPr_3)_2$ [1] and $Pb(HMDS)_2$[2] were prepared according to the reported procedure.

Preparation of complex 2.

Complex $OsH_6(P^iPr_3)_2$ (49 mg, 0.095 mmol) and $Pb(HMDS)_2$ (100 mg, 0.189 mmol) were dissolved in 1.5 mL of toluene in a schlenk and heated at 80 ºC for 22 h. The dark suspension formed was passed by a celite pad under argon. The filtrate was evaporated to dryness. Pentane (0.5 mL) was added to the crude and then evaporated to dryness several times until afford a brown solid. Crystals suitable for X-ray diffraction analysis were obtained from a concentrated solution of the solid in pentane at 243 K in the freezer of the glovebox. Yield: 66 mg (56%). Anal. Calcd. for $C_{36}H_{92}Os_2P_4Pb$: C, 34.96; H, 7.50. Found: C, 34.86; H, 7.45. HRMS (electrospray, *m/z*): Calcd. for $C_{36}H_{92}Os_2P_4Pb$ $C_{36}H_{92}Os_2P_4Pb$ $[M]^+$: 1240.5140, found: 1240.5099. IR (cm^{-1}): ʋ(Os-H) 2056(m), 1929(m) 1824(m). ^{1}H NMR (400 MHz, toluene-d_8, 298 K): δ 1.68 (sept, $^{3}J_{H\text{–}H} = 6.7$, 12H, CH(CH_3)$_2$), 1.20 (dvt, $^{3}N_{H\text{–}P} = 13.1$, $^{3}J_{H\text{–}H} = 7.0$, 72H, PCH(CH_3)$_2$), -8.07 (t, $^{2}J_{H\text{-}P} = 14.3$, 8H, OsH_4). T_1(min) (ms, 400 MHz, methylcyclohexane-d_{14}, 253 K): 239 ± 24. $^{31}P\{^{1}H\}$ NMR (162 MHz, toluene-d_8, 298 K): δ 55.7 (s). $^{13}C\{^{1}H\}$-APT NMR (101 MHz, toluene-d_8, 253 K): 32.1 (t, $^{1}J_{C\text{-}P} = 13.2$, CH(CH_3)$_2$), 20.7 (s, CH(CH$_3$)$_2$.

Structural Analysis of Complex 2

X-ray data were collected on a D8 Venture Bruker diffractometer (Mo radiation, λ = 0.71073 Å). The crystals were mounted under oil in a MiTeGen mount and cooled to 100(2) K with an open-flow nitrogen gas based cryostat (Oxford Cryosystems). Data were collected using φ and/or ω narrow scans. Diffracted intensities were integrated and corrected for absorption effects using SAINT[3] and SADABS[4] programs, included in APEX4 package.[3] The structure was solved by direct methods and refined by full-matrix least squares on F^2 with SHELXL2019,[5] including isotropic and subsequently anisotropic displacement parameters. The hydrogen atoms were observed in the last Fourier Maps or calculated, and refined freely or using a restricted riding model. The hydride ligands were observed in the Fourier maps of the last cycles of refinement and refined with restrained osmium hydride distances (d(Os-H)=1.59 Å).

Crystal data for **2**: $C_{36}H_{92}Os_2P_4Pb$, M_W 1236.56, yellow, irregular block, (0.095 x 0.074 x 0.033 mm^3), monoclinic, space group C2/c, *a*: 19.5209(7) Å, *b*: 13.0915(5) Å, *c*: 19.4859(7) Å, *β*: 110.5847(13)°, V = 4661.8(3) $Å^3$, Z = 4, Z' = 0.5, D_{calc}: 1.762 g cm^{-3}, F(000): 2408, T = 100(2) K, μ 9.199 mm^{-1}. 97474 measured reflections (2θ: 3-57°, ω and φ scans 0.5°), 5813 unique (R_{int} = 0.0499); min./max. transm. Factors 0.600/0.746. Final agreement factors were R^1 = 0.0263 (5200 observed reflections, I > 2σ(I)) and wR^2 = 0.0682; data/restraints/parameters 5813/4/220; GoF = 1.081. Largest peak and hole 2.013 (close to Os atoms) and -2.620 e/ $Å^3$.

NMR spectra

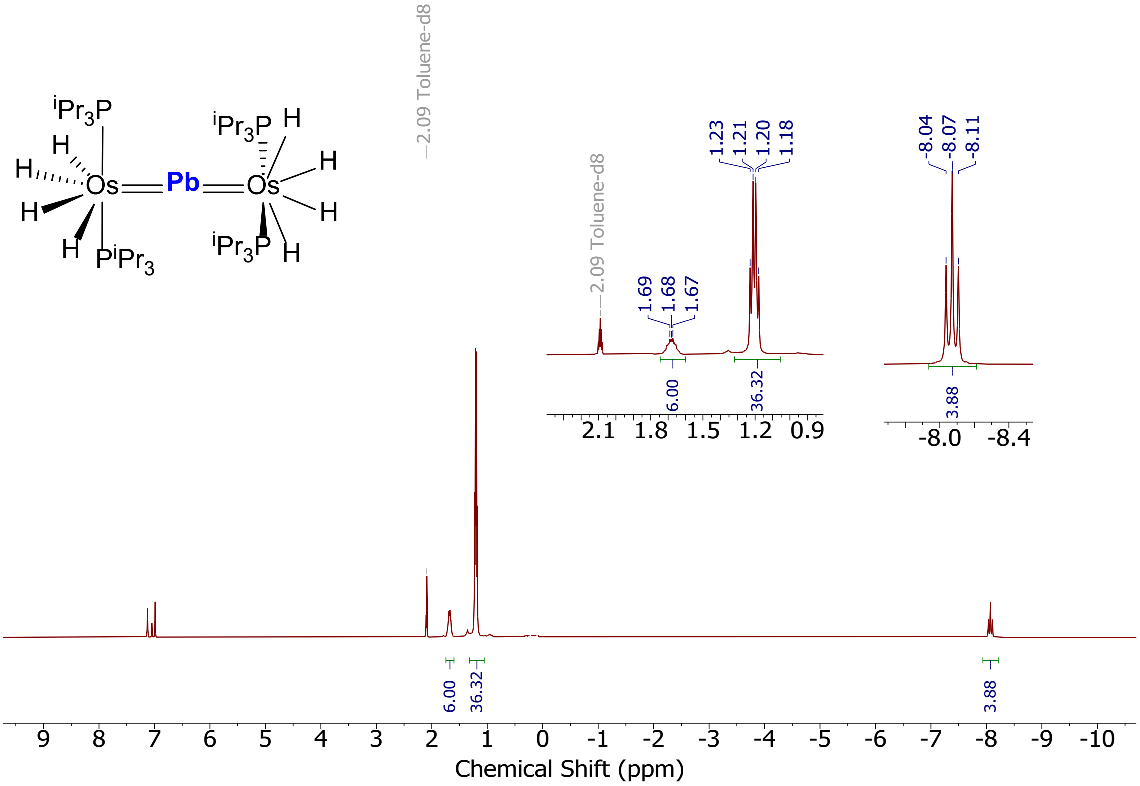

Figure S1. ^{1}H-NMR (400 MHz, toluene-d_8, 298 K) of complex **2**.

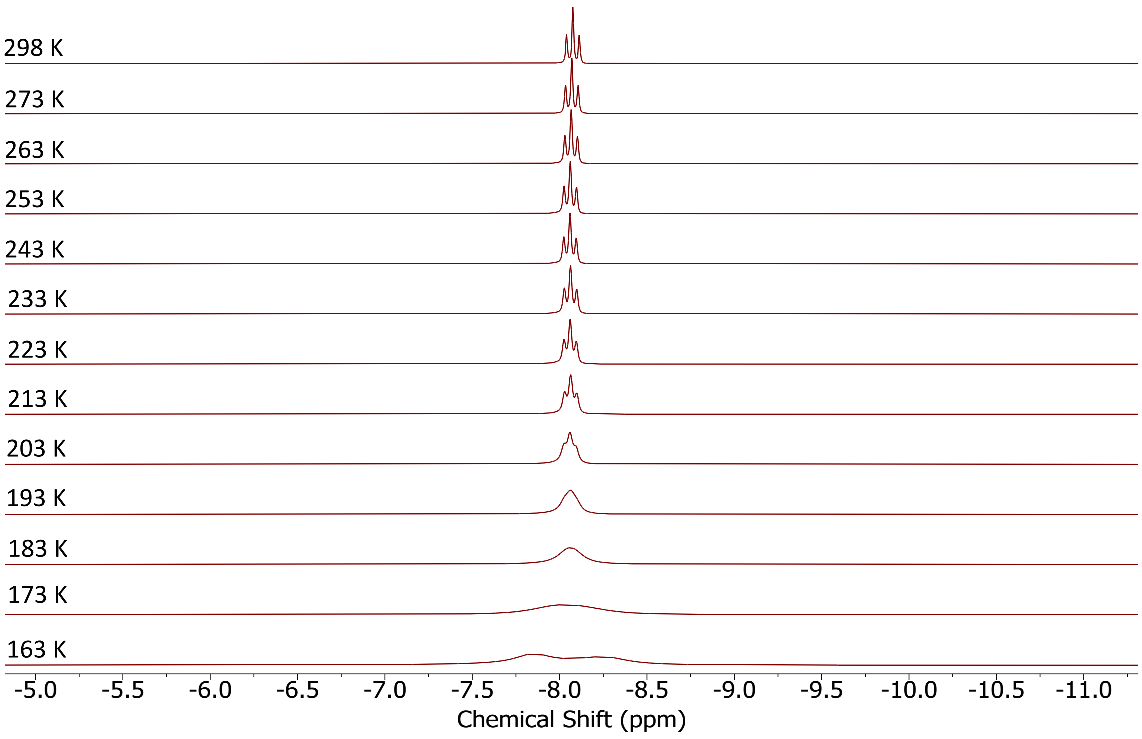

Figure S2. ^{1}H NMR as a function of the temperature (400 MHz, methylcyclohexane-d_{14}) of complex **2**.

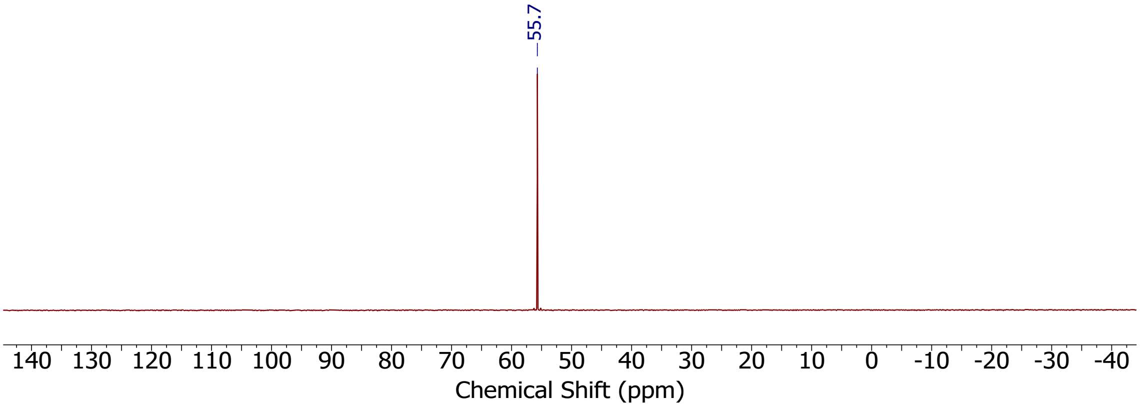

Figure S3. $^{31}P\{^{1}H\}$ NMR (162 MHz, toluene-d_8, 298 K) of complex **2**.

a)

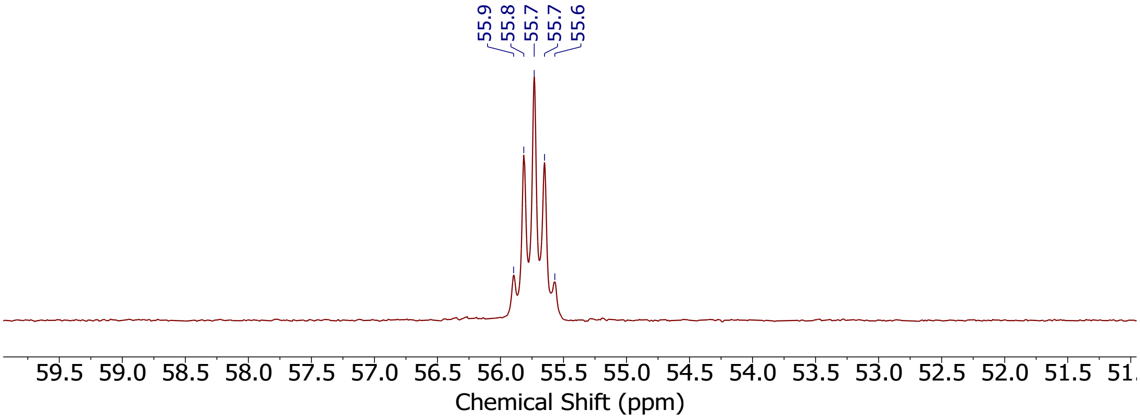

b)

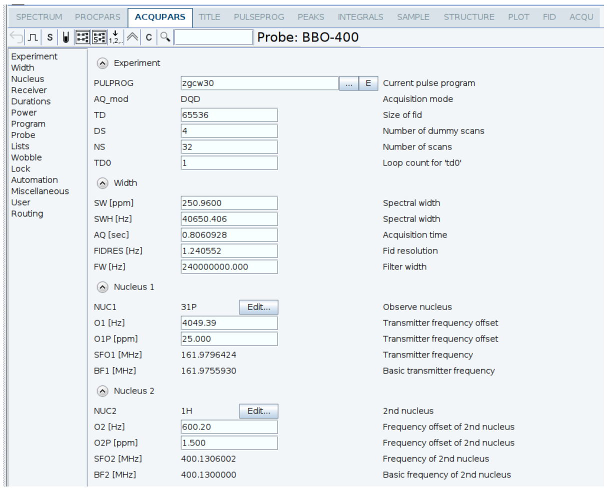

Figure S4. a) ^{31}P off-resonance NMR (162 MHz, toluene-d_8, 298 K) of complex **2** and b) adquisition parameters.

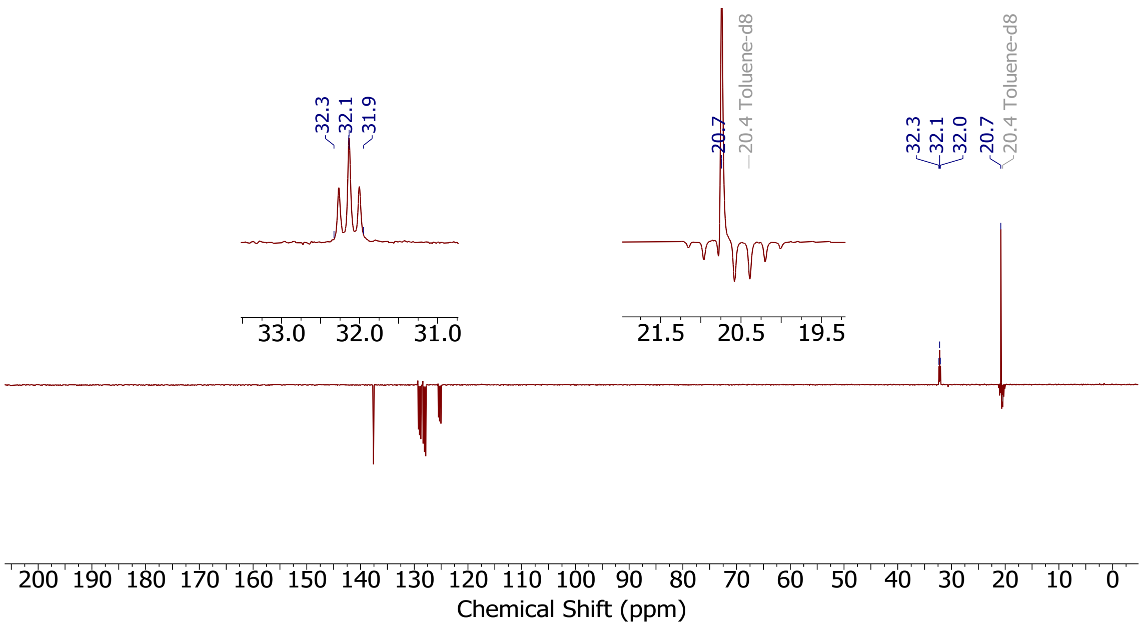

Figure S5. $^{13}C\{^1H\}$-APT NMR (100 MHz, toluene-d_8, 253 K) of complex **2**.

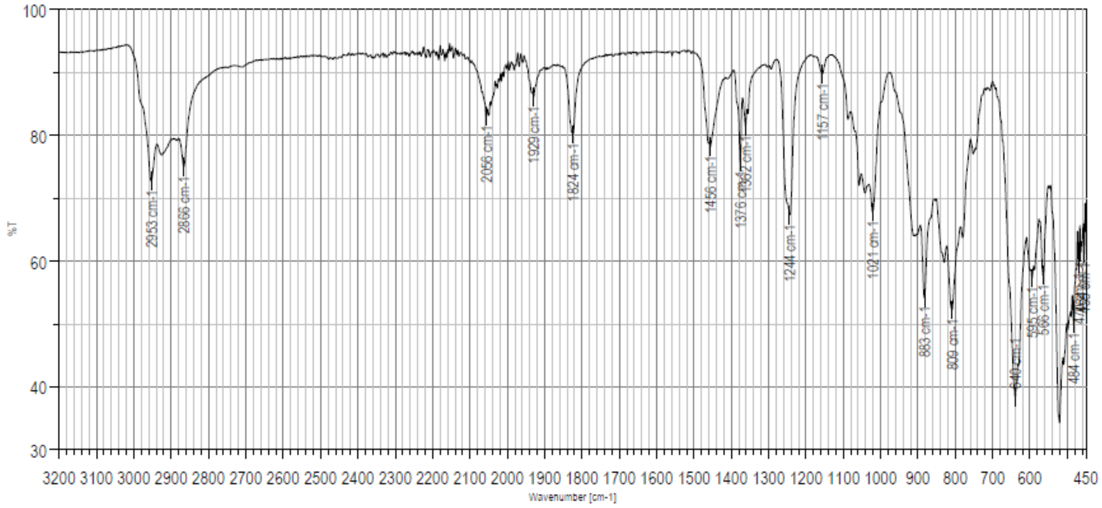

Figure S6. IR of complex **2**.

Theoretical Calculations

Geometry optimizations of the complexes were performed without symmetry constraints using the Gaussian09[6] optimizer together with Turbomole 7.1[7] energies and gradients at the BP86[8]/def2-TZVP[9] level of theory using the D3 dispersion correction suggested by Grimme et al.[10] and the resolution-of-identity (RI) approximation.[11] This level is denoted RI-BP86-D3/def2-TZVPP. Vibrational analysis was performed to ensure that the optimized geometry corresponds to an energy minimum.

The interaction ΔE_{int} between the selected fragments is analyzed with the help of the Energy Decomposition Analysis (EDA) method.[12] Within this approach, ΔE_{int} can be decomposed into the following physically meaningful terms:

$$\Delta E_{int} = \Delta E_{elstat} + \Delta E_{Pauli} + \Delta E_{orb} + \Delta E_{disp}$$

The term ΔE_{elstat} corresponds to the classical electrostatic interaction between the unperturbed charge distributions of the deformed reactants and is usually attractive. The Pauli repulsion ΔE_{Pauli} comprises the destabilizing interactions between occupied orbitals and is responsible for any steric repulsion. The orbital interaction ΔE_{orb} accounts for electron-pair bonding, charge transfer (interaction between occupied orbitals on one moiety with unoccupied orbitals on the other, including HOMO–LUMO interactions), and polarization (empty-occupied orbital mixing on one fragment due to the presence of another fragment). Finally, the ΔE_{disp} term takes into account the interactions which are due to dispersion forces. Moreover, the NOCV (Natural Orbital for Chemical Valence)[13] extension of the EDA method has been also used to further partition the ΔE_{orb} term. The EDA-NOCV approach provides pairwise energy contributions for each pair of interacting orbitals to the total bond energy.

The program package AMS 2020.101[14] was used for the EDA-NOCV calculations at the same BP86-D3 level, in conjunction with a triple-ζ-quality basis set using uncontracted Slater-type orbitals (STOs) augmented by two sets of polarization functions with a frozen-core approximation for the core electrons.[15] Auxiliary sets of s, p, d, f, and g STOs were used to fit the molecular densities and to represent the Coulomb and exchange potentials accurately in each SCF cycle.[16] Scalar relativistic effects were incorporated by applying the zeroth-order regular approximation (ZORA).[17] This level of theory is denoted ZORA-BP86-D3/TZ2P//RI-BP86-D3/def2-TZVP.

Total energies (in a. u., ZPVE included) of all the stationary points described in the text (RI-BP86-D3/def2-TZVP): **2:** E= -3167.4581535

References

(1) Aracama, M.; Esteruelas, M. A.; Lahoz, F. J.; López, J. A.; Meyer, U.; Oro, L. A.; Werner, H. Synthesis, Reactivity, Molecular Structure, and Catalytic Activity of the Novel Dichlorodihydridoosmium(IV) Complexes $OsH_2Cl_2(PR_3)_2$ (PR_3 = P-i-Pr_3, PMe-t-Bu_2). *Inorg. Chem.* **1991**, *30*, 288-293

(2) Cabeza, J. A.; Reynes, J. F.; García, F.; García-Álvarez, P.; García-Soriano, R. Fast and scalable solvent-free access to Lappert's heavier tetrylenes $E\{N(SiMe_3)_2\}_2$ (E = Ge, Sn, Pb) and $ECl\{N(SiMe_3)_2\}$ (E = Ge, Sn). *Chem. Sci.,* **2023**, **14**, 12477-12483

(3) SAINT+, version 6.01: Area-Detector Integration Software, Bruker AXS, Madison,WI, 2001

(4) Blessing, R. H. *Acta Crystallogr.* **1995**, *A51*, 33. SADABS: Area-detector absorption correction; Bruker- AXS, Madison, WI, 1996.

(5) SHELXL-2019/1. Sheldrick, G. M. A short history of *SHELX. Acta Cryst.* **2008**, *A64*, 112-122.

(6) Gaussian 09, Revision E.01, Frisch, M. J.; Trucks, G. W.; Schlegel, H. B.; Scuseria, G. E.; Robb, M. A.; Cheeseman, J. R.; Scalmani, G.; Barone, V.; Mennucci, B.; Petersson, G. A.; Nakatsuji, H.; Caricato, M.; Li, X.; Hratchian, H. P.; Izmaylov, A. F.; Bloino, J.; Zheng, G.; Sonnenberg, J. L.; Hada, M.; Ehara, M.; Toyota, K.; Fukuda, R.; Hasegawa, J.; Ishida, M.; Nakajima, T.; Honda, Y.; Kitao, O.; Nakai, H.; Vreven, T.; Montgomery, J. A., Jr.; Peralta, J. E.; Ogliaro, F.; Bearpark, M.; Heyd, J. J.; Brothers, E.; Kudin, K. N.; Staroverov, V. N.; Kobayashi, R.; Normand, J.; Raghavachari, K.; Rendell, A.; Burant, J. C.; Iyengar, S. S.; Tomasi, J.; Cossi, M.; Rega, N.; Millam, J. M.; Klene, M.; Knox, J. E.; Cross, J. B.; Bakken, V.; Adamo, C.; Jaramillo, J.; Gomperts, R.; Stratmann, R. E.; Yazyev, O.; Austin, A. J.; Cammi, R.; Pomelli, C.; Ochterski, J. W.; Martin, R. L.; Morokuma, K.; Zakrzewski, V. G.; Voth, G. A.; Salvador, P.; Dannenberg, J. J.; Dapprich, S.; Daniels, A. D.; Farkas, Ö.; Foresman, J. B.; Ortiz, J. V.; Cioslowski, J.; Fox, D. J. Gaussian, Inc., Wallingford CT, 2009.

(7) Ahlrichs, R.; Bär, M.; Häser, M.; Horn, H.; Kölmel C. Electronic structure calculations on workstation computers: The program system turbomole. *Chem. Phys. Lett.* **1989**, *162*, 165-169.

(8) (a) Becke, A. D. Density-functional exchange-energy approximation with correct asymptotic behavior. *Phys. Rev. A* **1988**, *38*, 3098-3100; (b) Perdew, J. Density-functional approximation for the correlation energy of the inhomogeneous electron gas. *P. Phys. Rev. B* **1986**, *33*, 8822-8824.

(9) Weigend, F.; Ahlrichs, R. Balanced basis sets of split valence, triple zeta valence and quadruple zeta valence quality for H to Rn: Design and assessment of accuracy. *Phys. Chem. Chem. Phys.* **2005**, *7*, 3297-3305.

(10) Grimme, S.; Antony, J.; Ehrlich, S.; Krieg, H. A consistent and accurate ab initio parametrization of density functional dispersion correction (DFT-D) for the 94 elements H-Pu. *J. Chem. Phys.* **2010**, *132*, 154104-19.

(11) Eichkorn, K.; Treutler, O.; Öhm, H.; Häser, M.; Ahlrichs, R. Auxiliary basis sets to approximate Coulomb potentials. *Chem. Phys. Lett.* **1995**, *242*, 652-660.

(12) For reviews on the EDA method, see: (a) Bickelhaupt, F. M.; Baerends, E. J. in Reviews in Computational Chemistry, (Eds. K. B. Lipkowitz, D. B. Boyd), Wiley-VCH: New York, 2000, Vol. 15, pp. 1-86; (b) von Hopffgarten, M.; Frenking, G. Energy decomposition analysis. *WIREs Comput. Mol. Sci.* **2012**, *2*, 43-62.

(13) Mitoraj, M. P.; Michalak, A.; Ziegler, T. A Combined Charge and Energy Decomposition Scheme for Bond Analysis. *J. Chem. Theory Comput.* **2009**, *5*, 962-975.

(14) ADF2020, SCM, Theoretical Chemistry, Vrije Universiteit, Amsterdam, The Netherlands, http://www.scm.com.

(15) Snijders, J. G.; Vernooijs, P.; Baerends, E. J. At. *Data Nucl. Data Tables* **1981**, *26*, 483-574.

(16) Krijn, J.; Baerends, E. J. Fit Functions in the HFS-Method, Internal Report (in Dutch), Vrije Universiteit Amsterdam, The Netherlands, 1984.

(17) (a) van Lenthe, E.; Baerends, E. J.; Snijders, J. G. Relativistic regular two-component Hamiltonians. *J. Chem. Phys.* **1993**, *99*, 4597-4610; (b) van Lenthe, E.; Baerends, E. J.; Snijders, J. G. Relativistic total energy using regular approximations. *J. Chem. Phys.* **1994**, *101*, 9783-9792; (c) van Lenthe, E.; Ehlers, A.; Baerends, E. J. Geometry optimizations in the zero order regular approximation for relativistic effects. *J. Chem. Phys.* **1999**, *110*, 8943-8953.
